# PROM at term: when might be the best time to induce labour? A retrospective analysis

**DOI:** 10.1007/s00404-025-07981-0

**Published:** 2025-03-29

**Authors:** Teresa Starrach, Lucia Ehmann, Hannah Volkmann, Andreas Flemmer, Anna Hester, Eileen Tremmel, Susanne Beyer, Linda Hertlein, Christoph Hübener, Roman Hornung, Thomas Kolben, Sven Mahner, Johanna Büchel

**Affiliations:** 1 Department of Obstetrics and Gynaecology, University Hospital Munich, Ludwig-Maximilians University Munich, Marchioninistr. 15, 81377 Munich, Germany; 2 Department of Neonatology, Dr. Von Hauner Children’s Hospital, Ludwig-Maximilians University Munich, Munich, Germany; 3 Department of Obstetrics and Gynaecology, University Hospital Würzburg, Würzburg, Germany; 4 Institute for Medical Information Processing, Biometry, and Epidemiology, Ludwig-Maximilians University Munich, Munich, Germany

**Keywords:** Term prelabour rupture of membranes, Induction of labour, Risk of infection, Postpartum infection-triple I, Chorioamnionitis, Expectant, Timing

## Abstract

**Purpose:**

PROM after 37 weeks of gestation occurs in approximately 10% of pregnancies. When spontaneous onset of labour does not follow, induction is recommended to decrease the risk of infection for both mother and child. However, there is no clear consensus on whether induction before 24 h after PROM results in fewer complications compared to induction after > 24 h.

**Material and methods:**

This retrospective observational study analysed the outcomes of 3174 women with PROM admitted to the delivery room of LMU Women's Hospital between 10/2015 and 09/2020. We evaluated whether timing of labour induction was associated with maternal or newborn postpartum infection rates.

**Results:**

Comparing women with spontaneous onset of labour to those who underwent induction, no significant differences were found in maternal CRP or leukocyte levels, fever, endometritis, or Group B streptococcus colonization. However, intrapartum antibiotic therapy was significantly higher in the induction group. When the induction group was subdivided based on the interval from PROM to induction, no significant differences were observed in maternal infection parameters, need for antibiotics, postpartum length of hospital stay, or endometritis. For newborn infections, a significant difference in CRP levels was found, with higher levels in the groups with “induction < 12 h” and “> 24 h”.

**Conclusion:**

The presented data suggests that waiting for spontaneous contractions within the first 24 h after PROM was not associated with the risk of infection if no initial signs for infection are present. However, beyond 24 h, the risk of infection increased. These findings support current recommendations regarding the timing of induction after PROM.

## What does this study add to the clinical work

**Table:** 

As PROM at term is common, it is important to discuss the correct approach. The presented data suggests that, in patients with normal infection levels, waiting for spontaneous onset of labour up to 24 hours is not associated with a significantly increased risk of infection for mother and child. The rate of patients who develop spontaneous labour and therefore do not require induction may be increased without taking any major risks.	

## Introduction

Prelabour or premature rupture of membranes (PROM) at term is defined as the rupture of the amniotic membranes before the onset of labour or regular uterine contractions at ≥ 37 + 0 weeks of gestation. It occurs in approximately 10% of pregnancies [6, 8, 16]. After PROM, spontaneous labour begins within 24 h in around 60% of cases, and within 72 h in over 95% of cases [9, 11, 13]. However, as the interval between membrane rupture and birth increases, so does the risk of infection for both mother and child [13]. The optimal duration of expectant management, without significant increasing in the peripartum complication rate, remains unclear.

In addition to the time interval between PROM and delivery, the risk of infection is influenced by several factors: the number of vaginal examinations before delivery, the duration of active labour, the presence of meconium-stained amniotic fluid, colonization with group B streptococci, and the latency interval before the onset of labour [18]. However, the duration of the latency interval itself appears to be an independent risk factor for clinical chorioamnionitis [18].

A systematic review by Middleton et al. compared immediate or early intervention (within 24 h) with expectant management in women with PROM at term [16]. Active management was associated with a shorter interval to labour onset (− 10 h, 95% CI − 12 to -8 h), a lower rate of amniotic infection syndrome (AIS) and/or endometritis, fewer neonatal transfers to paediatric hospital or neonatal intensive care unit (NICU), and a slight, but non-significant reduction in early-onset neonatal sepsis compared to the expectant management. Additionally, patients tended to be more satisfied with active management. However, there were no significant differences in caesarean delivery rates or perinatal mortality. It is important to note that the overall quality of the included studies was rated as low [16].

The German guideline on labour induction recommends active management at least 24 h after PROM, although it does not specify an exact time frame for induction [14].

However, whether even earlier labour induction—at 6, 12, or 18 h after PROM – offers additional benefits remains unclear. No data currently exists regarding the impact of earlier active management on infection risk. This retrospective analysis aimed to determine whether the risk for maternal and neonatal infections decreases with earlier labour induction after PROM.

The hypothesis underlying the study posits that earlier induction of labour is associated with a reduced incidence of fetal and maternal infections, provided spontaneous onset of labour does not occur.

## Material and methods

### Study description

The objective of this study was to focus on maternal and neonatal infections, including sepsis postpartum. We analysed data from patients with PROM treated in one of the two perinatal centres of the LMU Women's Hospital using our clinical systems such as SAP and Viewpoint. The study covered a five-year period from October 2015 to September 2020.

This study was designed as a retrospective observational monocentric study and received ethical approval from the LMU Ethics Committee (No. 22-0569) on July 6, 2022. It was conducted in accordance with the standards of the Declaration of Helsinki (1975, revised in 2008). The trial was registered at the German Clinical Trials Register (DRKS) on July 18, 2022 (trial registration number DRKS00029411, URL https://drks.de/search/de/trial/DRKS00029411).

All personal patient data was fully anonymized: patient names were replaced with unique numerical identifiers, and the date of birth was encoded to prevent re-identification.

Inclusion criteria for the study were PROM at ≥ 37 weeks of gestation. Patients with multiple pregnancies, premature PROM before 37 weeks of gestation (PPROM), or planned caesarean section were excluded.

### Outcome measures

Basic demographic and clinical parameters recorded included gestational age, date of birth, newborn length and weight, as well as pregnancy-related risk factors.

To assess the level of infection, several laboratory and clinical parameters were evaluated. For mothers, leukocyte counts were recorded and classified as conspicuous if they were ≥ 15 G/l. C-reactive protein (CRP) levels were also measured and deemed conspicuous if they were ≥ 5 mg/dl. For newborns, CRP levels were evaluated, with values ≥ 0.5 mg/dl considered abnormal. If CRP was not measurable, a value < 0.1 was recorded. Since this value did not allow statistical evaluation, a CRP of 0.05 was used.

Additionally, interleukin-6 (IL-6) levels were measured, with values > 50 pg/ml classified as conspicuous. Further assessments included blood culture analysis.

Other clinical data collected encompassed the length of the mother’s hospital stay, whether the newborn required admission to an intensive care unit (ICU) or monitoring ward, and whether antibiotic treatment was administered to the mother and/or newborn. It was recorded whether B streptococci were detected. In this case, antibiotic treatment with Penicillin G was administered from the time of hospital admission. In women with negative or unknown B streptococcus status, prophylactic antibiotic treatment with Ampicillin/Sulbactam was started 18 h after PROM. In the case of elevated infection values antibiotic therapy was started immediately after PROM with Ampicillin/Sulbactam.

Neonatal outcomes were further quantified using the APGAR score and pH levels in the umbilical artery.

Additional data was collected concerning the induction of labor and the circumstances surrounding birth. Information on labor induction included the date and time of induction, the method employed, the time span between premature rupture of membranes (PROM) and induction, as well as the intervals from induction to birth and from PROM to birth.

Comprehensive birth-related data was recorded to provide insights into delivery outcomes and associated risks. This included the date and time of PROM and birth, the mode of delivery (e.g., vaginal or operative), and any associated birth risks. For cases involving operative delivery, the specific indications leading to this intervention were documented. The use of epidural anesthesia during labor was noted, as was the administration of antibiotics during the intrapartum period.

Additional parameters recorded included the presence of fever during labor, the occurrence of meconium-stained amniotic fluid, and whether the labor period was prolonged. Cardiotocographic (CTG) findings were analyzed, with abnormalities classified as suspect or pathologic based on FIGO score. Cases of diagnosed endometritis were also documented.

### Sample size calculation and statistical analysis

To ensure sufficient statistical power, a sample size calculation was performed with a significance level (α) of 0.05, power of 90%, and an expected prevalence (PROM) of 8–10%. The study included two groups (spontaneous labor and induction) in a 2:1 ratio. A chi-square test was planned to compare group proportions. The total required sample size was 3257 participants: 2201 in Group 1 and 1056 in Group 2, ensuring adequate power and a 5% type I error rate. The required numbers were approximately achieved through the analysis of the aforementioned 5-year period.

Statistic evaluation was conducted in cooperation with the LMU Institute for Medical Information Processing, Biometry, and Epidemiology (IBE), using Windows Excel and SPSS Statistics 28. To analyse the data non-parametric statistical methods were used, as continuous variables were not normally distributed. Continuous variables are presented as mean values with standard deviations, while categorical variables are described as counts and percentages. A multivariate analysis was performed to identify risk factors, such as maternal age, BMI and other maternal or neonatal factors which could lead to worse perinatal outcomes. The significance level was set at 5%, with *p* < 0.05 considered statistically significant. Given the number of statistical tests conducted, *p* values were adjusted for multiple comparisons to reduce the risk of false-positive results. For this purpose, we applied the Bonferroni-Holm correction for multiple testing.

## Results

### Collective

Between October 2015 and September 2020, a total of 19,423 children were born at the Department of Obstetrics and Gynaecology, LMU hospital, across both the Campus Innenstadt and Campus Großhadern. During this period, 3174 pregnant women (16.3%) experienced PROM at term and were included in this analysis. Among the 3174 women with PROM, 69.2% (n = 2195) developed spontaneous labour, while 30.8% (n = 977) required induction of labour. Two cases could not be reliably categorized regarding whether induction took place or not and were thus excluded from the analysis.

**Fig. 1:**
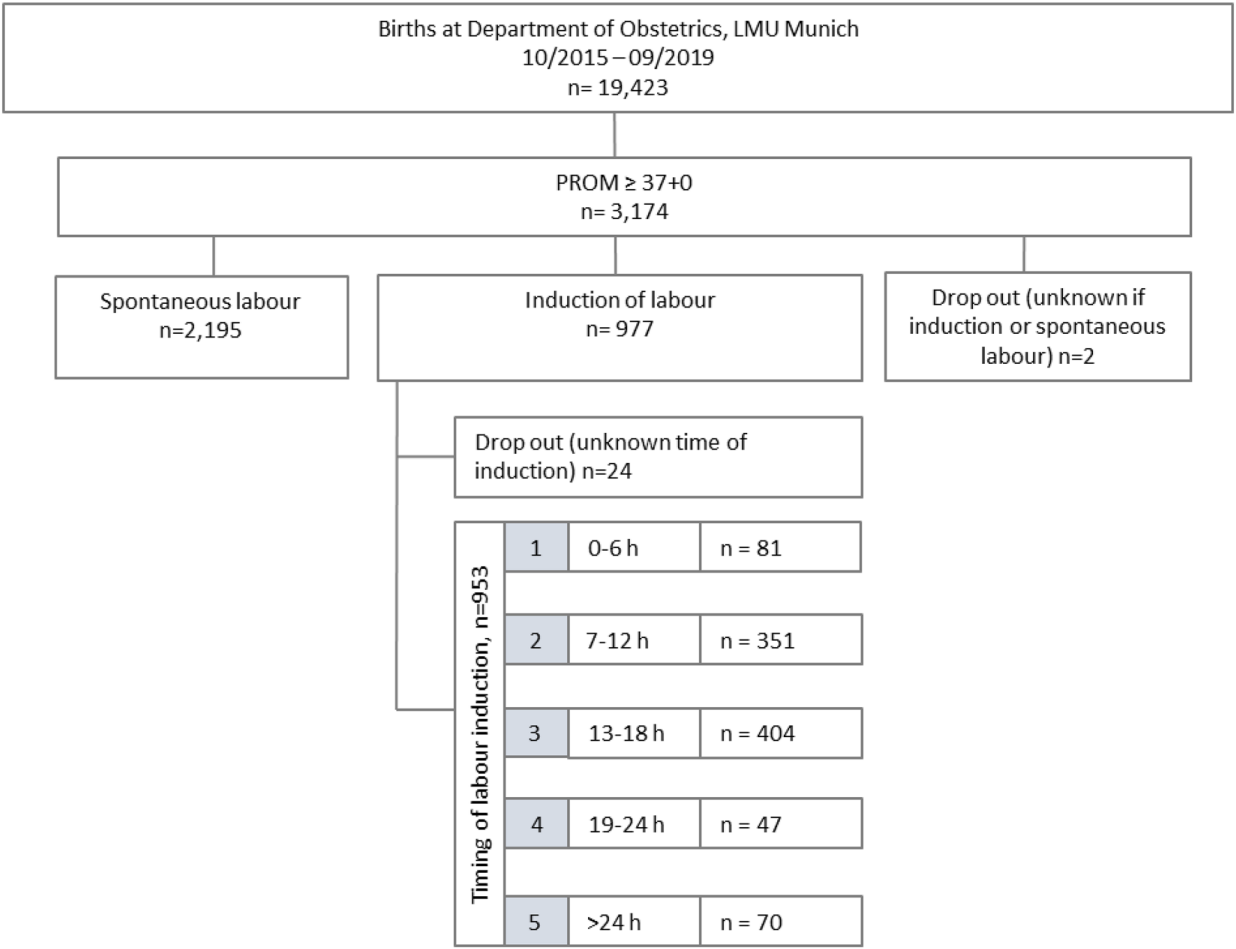
The collective and its division into groups according to time until induction of labour or spontaneous onset of labour and the time from prom until induction of labour

The women who underwent induction of labour were further divided into five groups based on the time interval between PROM and the initiation of labour induction: Group 1 included those induced within 0–6 h after PROM, Group 2 within > 6–12 h, Group 3 within > 12–18 h after PROM, Group 4 within > 18–24 h and Group 5 after 24 h. For 24 women, data on the initiation time was missing, and they were consequently excluded from further analysis (Fig. 1).

### Maternal and neonatal infection with or without induction of labour

Focusing on maternal infections, 65.5% (n = 2077) of all mothers with PROM ≥ 37 gestational weeks did not have elevated infection markers either before or after birth. In the spontaneous labour group, 34.6% of women presented with elevated infection markers before or after labour, compared to 34.5% in the induction group. The median leukocyte counts were similar in both groups, with 13.3 G/l in the spontaneous labour group and 13.5 G/l in the labour induction group; in 111 cases (3.5%) leukocyte levels were missing. The median CRP was 4.4 mg/dl in women without intervention and 3.9 mg/dl in women who underwent labour induction, with no significant differences observed between the two groups. Similarly, no significant differences were found regarding postpartum antibiotic therapy, postpartum diagnosis of endometritis, GBS status, meconium-stained amniotic fluid, or fever during labour. However, a significant difference was observed in the rate of intrapartum antibiotic use: 38.3% in the spontaneous labour group vs. 70.3% in the induction group (*p* = 0.046). Specific results are shown in Table 1.

**Table 1:** Maternal infection with or without induction of labour

	PROM ≥ 37 + 0 n = 3172	Spontaneous onset of labour, n = 2195	Induction of labour, n = 953	*p* value
CRP in mg/dl (Median and CI 95%)	4.3 (3.4–5)	4.4 (3.15–5.11)	3.9 (2.6–6.6)	> 0.99
Leukocytes in G/l (Median and CI 95%)	13.4 (13.2–13.5)	13.3 (13.2–13.5)	13.5 (13.2–13.8)	> 0.99
Elevated infection parameters*	1196 (34.6%)	759 (34.6%)	337 (34.5%)	> 0.99
Antibiotic therapy intrapartum	1523 (48.1%)	839 (38.3%)	684 (70.3%)	**0.046**
Antibiotic therapy postpartum	183 (7.6%)	127 (7.9%)	56 (7.0%)	> 0.99
Fever intrapartum	164 (5.2%)	104 (4.7%)	60 (6.1%)	> 0.99
Meconium-stained amniotic fluid	331 (10.4%)	237 (10.8%)	94 (9.6%)	> 0.99
Diagnosis of endometritis postpartum	11 (0.3%)	9 (0.4%)	2 (0.2%)	> 0.99
Group B streptococcus colonization	351 (15.2%)	239 (15.0%)	112 (15.6%)	> 0.99

^*^Elevated infection levels: leukocytes ≥ 15 G/l and/or CRP ≥ 5 mg/dl. Significant results are shown in bold

Regarding the infection parameters after birth, 558 (17.6%) neonates showed elevated infection parameters in serum. Routine blood sampling was not performed in clinically unremarkable children, resulting in undetermined CRP levels in 1204 (37.9%) and undetermined IL-6 levels in 1236 (38.9%) babies. A total of 556 (17.6%) newborns were admitted to NICU or a monitoring ward and 11.2% (n = 350) received antibiotics after birth. If a blood culture was performed, it was positive in 5.5% (18/325). When dividing the neonates into two groups depending on whether their mothers underwent labour induction, 83.3% (1826/2192) of the neonates in the spontaneous labour group had no elevated infection parameters. Conversely, 16.7% of infants in the spontaneous labour group had elevated infection levels, compared to 19.7% in the induced labour group. This difference is no longer significant after adjustment for multiple testing. Similarly, there were no significant differences between the groups with regards to postnatal antibiotic therapy and blood culture results. In the induction group, 19.8% (n = 193) of neonates had to be admitted to NICU or monitoring ward, in contrast to only 16.5% (n = 363) in the group without induction of labour, showing a trend without reaching statistical significance (Table 2).

**Table 2:** Neonatal infection with or without induction of labour

	All n = 3172	Spontaneous onset of labour n = 2195	Induction of labour n = 953	*p* value	not tested
CRP in mg/dl (Median and CI 95%)	0.05 (0.05–0.05)	0.05 (0.05–0.05)	0.05 (0.05–0.05)	0.775	1206 (38.0%)
IL-6 in pg/ml (Median and CI 95%)	15.9 (15–16.8)	16.2 (15–17.7)	15.3 (13.9–16.6)	> 0.99	1268 (39.9%)
Elevated infection parameters*	558 (17.6%)	366 (16.7%)	192 (19.7%)	> 0.99	6 (0.19%)
Postnatal antibiotic therapy	350 (11.2%)	228 (10.5%)	122 (12.6%)	> 0.99	46 (1.4%)
Admission to NICU	556 (17.5%)	363 (16.5%)	193 (19.8%)	0.84	5 (0.16%)
Positive blood cultures	18 (5.5%)	10 (4.4%)	8 (7.8%)	> 0.99	2847 (89.7%)

^*^Elevated infection parameters: CRP ≥ 5 mg/dl and/or IL-6 ≥ 50 pg/ml

### Maternal and neonatal infection according to time to induction

Five groups were established based on the timing of labour induction following PROM (Fig. 1). These groups were compared regarding maternal infection parameters, maternal antibiotic therapy during and after labour, intrapartum fever, meconium-stained amniotic fluid, postpartum diagnosis of endometritis, and GBS status. After adjusting the *p* value for multiple testing, two significant differences emerged between the groups in relation to these parameters (Table 3): postpartum antibiotic administration was significantly more frequent in the group where labour was induced within 0–6 h compared to all other groups (26.5% vs. 5.1–7.8%, *p* = 0.046). It should be noted that in 164 cases (17.2%), data on whether antibiotic therapy was administered were missing.

**Table 3:** Maternal infection according to time to induction

	Induction of labour n = 953	Group 1: 0–6 h n = 81	Group 2 > 6–12 h n = 351	Group 3 > 12–18 n = 404	Group 4: > 18–24 n = 47	Group 5: > 24 h n = 70	*p* value
CRP in mg/dl (Median and CI 95%)	3.9 (2.6–6.6)	3.6 (1.9–8.2)	3.7 (1.8–7.4)	5.1 (1.8–11.2)	1 (0.5–3.9)	7.2 (3.0–8.5)	> 0.99
Leukocytes in G/l, (Median and CI 95%)	13.5 (13.2–13.8)	13.6 (12.8–14.9)	13.4 (13.0–13.8)	13.6 (12.9–13.9)	13.4 (12.4–14.7)	13.1 (11.6–14.2)	> 0.99
Elevated infection parameters*	326 (34.2%)	35 (43.2%)	118 (33.7%)	135 (33.4%)	14 (29.8%)	24 (34.3%)	> 0.99
Antibiotic therapy intrapartum	664 (70.0%)	28 (35.0%)	193 (55.5%)	341 (84.4%)	40 (85.1%)	62 (88.6%)	**0.046**
Antibiotic therapy postpartum	56 (7.0%)	9 (26.5%)	20 (7.8%)	20 (5.1%)	3 (7.1%)	4 (6.3%)	**0.046**
Fever intrapartum	56 (5.9%)	9 (11.1%)	26 (7.4%)	16 (4.0%)	0 (0.0%)	5 (7.1%)	0.736
Meconium-stained amniotic fluid	90 (9.4%)	12 (14.8%)	26 (7.4%)	37 (9.2%)	6 (12.8%)	9 (12.9%)	> 0.99
Diagnosis of endometritis postpartum	2 (0.2%)	0 (0.0%)	2 (0.6%)	0 (0.0%)	0 (0.0%)	0 (0.0%)	> 0.99
Group B streptococcus colonization	108 (11.3%)	6 (7.4%)	51 (14.5%)	43 (10.6%)	1 (2.1%)	7 (10.0%)	> 0.99

Group 1: induction 0–6 h after PROM; Group 2: induction > 6–12 h after PROM; Group 3: induction > 12–18 h after PROM; Group 4: induction > 18–24 h after PROM; Group 5: induction > 24 h after PROM

^*^Elevated infection parameters: leukocytes ≥ 15 G/l and/or CRP ≥ 5 mg/dl. Significant results are shown in bold

Another significant difference concerned antibiotic administration during labour: 35% of women in group 1 received antibiotics, compared to 55.5% in group 2 and over 80% in groups 3–5 (*p* = 0.046). Data was missing for only four women (0.4%) in this analysis. Intrapartum fever was most common in group 1 (11.1%) and least common in group 4 (0%, *p* = 0.736). All maternal values are detailed in Table 3.

As a next step, various parameters of newborns whose mothers underwent induction of labour were examined in greater detail and analysed for differences based on the timing of induction following PROM. Of the 952 infants included, 746 blood samples were collected for CRP measurement and 737 for IL-6 measurement. Routine blood sampling was not performed in clinically normal infants.

**Fig. 2:**
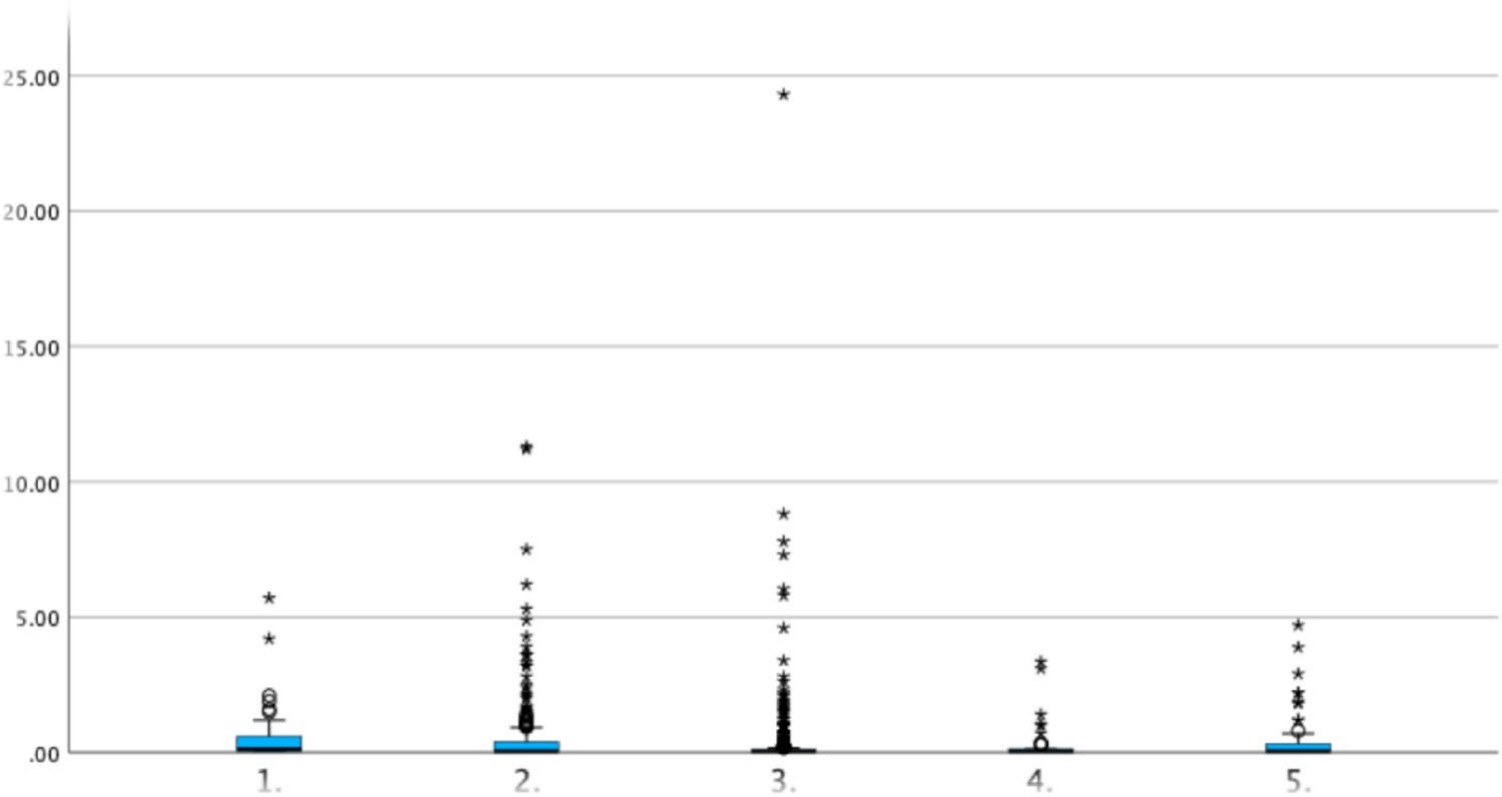
Children’s CRP levels in mg/l (x-axis) according to the groups of time of induction (y-axis). Group 1: induction within 0–6 h after PROM. Group 2: induction within > 6–12 h after PROM. Group 3: induction within > 12–18 h after PROM, Group 4: induction within > 18–24 h after PROM, Group 5: induction > 24 h after PROM

A statistically significant difference was observed in the highest measured CRP values among the newborns. The median CRP level was slightly higher in infants whose mothers underwent induction within 0–6 h after PROM (0.13 mg/dl) compared to all other induction groups (0.05 mg/dl, *p* = 0.046, Table 4 and Fig. 2). As written in the methods section, it is important to note that values below 0.1 are recorded when CRP is undetectable. As such values are not amenable to statistical analysis, a CRP value of 0.05 was assigned.

**Table 4:** Neonatal infection according to time to induction

	Induction of labour n = 953	Group 1: 0–6 h n = 81	Group 2: > 6–12 h n = 351	Group 3: > 12–18 n = 404	Group 4: > 18–24 h n = 47	Group 5: > 24 h n = 70	*p* value
CRP in mg/dl (Median and CI 95%)	0.05 (0.05–0.05)	0.13 (0.05–0.35)	0.05 (0.05–0.05)	0.05 (0.05–0.05)	0.05 (0.05–0.05)	0.05 (0.05–0.08)	**0.046**
IL-6 in pg/ml (Median and CI 95%)	15.20 (13.9–16.6)	38.25 (20.4–58.1)	15.20 (12.9–19.4)	13.90 (13.1–16.2)	14.50 (11.6–23.3)	15.95 (11.9–23.0)	0.165
Elevated infection parameters	184 (19.3%)	24 (29.6%)	60 (17.1%)	71 (17.6%)	13 (27.7%)	16 (22.9%)	0.17
Antibiotic therapy postpartum	119 (12.6%)	13 (17.1%)	41 (11.8%)	46 (11.5%)	8 (17.0%)	11 (15.7%)	> 0.99
Admission to NICU	188 (19.7%)	18 (22.2%)	69 (19.7%)	73 (18.1%)	14 (29.8%)	14 (20.0%)	> 0.99
Positive blood cultures	8 (8.0%)	1 (8.3%)	1 (2.4%)	3 (10.0%)	2 (28.6%)	1 (10.0%)	> 0.99

Group 1: induction within 0–6 h after PROM; Group 2: induction within > 6–12 h after PROM; Group 3: induction within > 12–18 h after PROM; Group 4: induction within > 18–24 h after PROM; Group 5: induction > 24 h after PROM

Elevated infection parameters: CRP ≥ 5 mg/dl and/or IL-6 ≥ 50 pg/ml. Significant results are shown in bold

The median IL-6 values followed a similar, though non-significant, trend: the median IL-6 level was slightly higher in induction group 1 (38.25 pg/ml) compared to the other groups (13.90–15.95 pg/ml, *p* = 0.17).

No significant differences were observed between the groups in terms of ICU admission, postnatal antibiotic therapy, or positive blood cultures.

### Influence of induction of labour on rates of caesarean section

87.8% (n = 2784) of all births occurred via vaginal or vacuum-assisted delivery, while 12.2% (n = 388) were delivered by caesarean section. Among women who did not require labour induction, 9.7% (n = 212) underwent a caesarean section, compared to 18% (n = 176) in the group of women who did undergo labour induction. This difference was statistically significant (*p* = 0.046, Table 5).

**Table 5:** Mode of birth with or without induction of labour

	PROM ≥ 37 + 0 n = 3172	Spontaneous onset of labour n = 2195	Induction of labour n = 953
Vaginal and vacuum-assisted delivery	2784 (87.8%)	1983 (90.3%)	801 (82.0%)
Caesarean section	388 (12.2%)	212 (9.7%)	176 (18.0%)

Statistically significant difference in mode of delivery between spontaneous onset of labour and labour induction (*p* = 0.046)

Subsequently, we examined the impact of induction timing on caesarean section rates. No statistically significant differences were observed between the induction timing groups (*p* > 0.99), indicating that caesarean section rates were comparable irrespective of the timing of induction in this study (Table 6).

**Table 6:** Caesarean section rate according to time of induction

	Induction of labour n = 953	Group 1: 0–6 h n = 81	Group 2 > 6–12 h n = 351	Group 3 > 12–18 n = 404	Group 4: > 18–24 n = 47	Group 5: > 24 h n = 70
Vaginal and vacuum-assisted delivery	791 (83.0%)	66 (81.5%)	290 (82.6%)	341 (84.4%)	37 (78.7%)	57 (81.4%)
Caesarean section	162 (17.0%)	15 (18.5%)	61 (17.4%)	63 (15.6%)	10 (21.3%)	13 (18.6%)

No statistically significant differences between the induction timing groups (*p* > 0.99)

Group 1: induction 0–6 h after PROM; Group 2: induction > 6–12 h after PROM; Group 3: induction > 12–18 h after PROM;

Group 4: induction > 18–24 h after PROM; Group 5: induction > 24 h after PROM

## Discussion

PROM at term leads to spontaneous labour in around 60% of cases within 24 h [13]. However, the risk of perinatal morbidity increases with the duration of time after PROM. German national guidelines recommend induction of labour no later than 24 h after PROM. The question remains whether earlier induction might offer additional benefits. This retrospective analysis investigates the association between the timing of labour induction and maternal and neonatal infections.

Of 19,423 women, 3174 (16.3%) pregnant women were identified with PROM ≥ 37 + 0 weeks of gestation. Abu Shqara describes a similar prevalence of 16.9% in a recently published retrospective study [1]. In the literature, a prevalence of 8–10% is frequently cited, in line with the findings of Cammu and Hannah from the 1990s [6, 13]. The reasons for the discrepancy in prevalence remain unclear. One possible explanation could be a general increase in the number of births starting with premature rupture of membranes in recent years. However, it cannot be ruled out that the cause lies in the retrospective study design. Inaccuracies in the documentation can neither be ruled out nor reliably verified in retrospect. Of the 3174 pregnant women with PROM, spontaneous labour occurred in 2195 women (69.16%) and labour was induced in 30.8%.

Comparison between women with spontaneous labour and those with induction revealed no significant differences in laboratory and clinical infection parameters for either the mothers or the newborns. The only significant difference was a higher rate of intrapartum antibiotic therapy in the induction group (*p* = 0.046). Furthermore, it is important to consider that during the time of this study, it was standard practice at LMU hospitals to administer antibiotics to patients experiencing PROM > 18 h without active labour.

When analysing the timing of induction, no significant differences were found in maternal infection parameters, postpartum endometritis, or meconium-stained amniotic fluid. Group 1 (induction 0–6 h after PROM) showed a tendency towards more frequent fever during labour and slightly higher maternal infection scores, though these differences were not statistically significant.

The newborns in group 1 (induction 0–6 h after PROM) had statistically significantly higher CRP levels, although this was only a very small clinical difference. Il-6 levels also tended to be higher in group 1 than in the other groups, but again the clinical difference was small. Overall, the data presented here shows neither an advantage nor a disadvantage with regard to the time of induction within the first 24 h after PROM. Existing studies vary greatly in design, methods, and timing of induction [7, 15]. There is consensus that both maternal and neonatal morbidity increase with longer intervals between PROM and delivery. International guidelines generally recommend labour induction after 24 h [2, 17]. The German Guideline suggests induction should be recommended no later than 24 h after PROM [14]. Recent studies and reviews have questioned whether earlier induction within 24 h might be beneficial [3, 4, 15].

In a secondary analysis of the TERMPROM study, Melamed et al. found that early induction within 15–20 h reduces neonatal and maternal risks compared to expectant management without increasing the risk of caesarean delivery [15]. Maternal and neonatal infection risks were assessed at 5-h intervals. A notable increase in infection rates was observed approximately 20 h after PROM, while infection rates remained similar across the 0–4, 5–9, and 10–14-h groups.

In our cohort, group 4 (18–24 h after PROM) did not exhibit worse maternal or neonatal outcomes or higher infection rates. However, the relatively small number of cases in this group (n = 47) should be noted. A recent review by Belussi et al. concluded that immediate induction after PROM is associated with a significantly lower maternal and infant morbidity than a wait-and-see approach. They are therefore convinced that immediate induction after PROM is the optimal treatment strategy [4]. Similarly, Bachar et al. found no difference in maternal infections but noted more newborns received antibiotics and had more adverse outcomes when induction was delayed beyond 24 h [3].

Our retrospective data does not support a higher rate of maternal or neonatal infections within the first 24 h after PROM. Slightly elevated infection scores were observed in women induced immediately after PROM (0–6 h), possibly because these women presented already with elevated infection scores or a higher risk to get an infection. It is also possible that a pre-existing infection led to PROM in this group. In the other groups, infection may have occurred later or not at all.

In our analysis, caesarean section rates were significantly higher among women who underwent labour induction compared to those who did not (18% vs. 9.7%, *p* = 0.046). However, caesarean section rates remained consistent regardless of the timing of labour induction.

The question of whether induction itself increases the risk of caesarean delivery is highly debated.

In the ARRIVE trial, no increased caesarean section rate was observed following labour induction in a low-risk population [10]. In contrast, Butler et al. reported a significant increase in caesarean deliveries associated with labour induction [5]. Here, in this retrospective study caesarean section rate was the primary endpoint (unlike the ARRIVE trial) [5]. Supporting our findings, a secondary analysis of the TERMPROM study also demonstrated no impact of induction timing on caesarean section rates [15].

While recent studies and reviews prefer immediate induction after PROM, pregnant women often wish for a natural, spontaneous start of labour. That is why some studies support a wait-and-see approach for up to 24 h even in outpatient setting without increased infection rates [12, 19]. Nevertheless, there is a 64% chance that labour will begin spontaneously within 24 h without intervention [15].

Historically, satisfaction with immediate induction was higher [13]. It is possible that attitudes towards labour induction have shifted in recent years. However, the data from the largest study available on term PROM should be included in the counselling of a pregnant woman.

This study has limitations due to its retrospective design. Only collected data could be analysed; infection diagnostics were only performed if medically indicated, leading to missing data of clinically unsuspicious mothers and newborns. Particularly, the smaller sample sizes in the > 18 h groups (n = 47 and n = 70 of 3172), may have influenced the results.

## Conclusions and outlook

Our data suggests that induction within 24 h after PROM does not result in higher infection rates, and therefore a wait-and-see approach in the first 24 h seems justifiable. However, based on available prospective studies, including the TERMPROM analyses, immediate induction after PROM should be considered. Further research is needed to assess patient satisfaction with different induction protocols and the economic implications of prolonged waiting.

## Data Availability

No datasets were generated or analysed during the current study.
